# Supplementary figures and images for: MHC class I H2-Kb negatively regulates neural progenitor cell proliferation by inhibiting FGFR signaling
Source: PLoS Biol. 2021 Jun 28;19(6):e3001311. doi: 10.1371/journal.pbio.3001311 (PMC8270425; doi:10.1371/journal.pbio.3001311)

**A**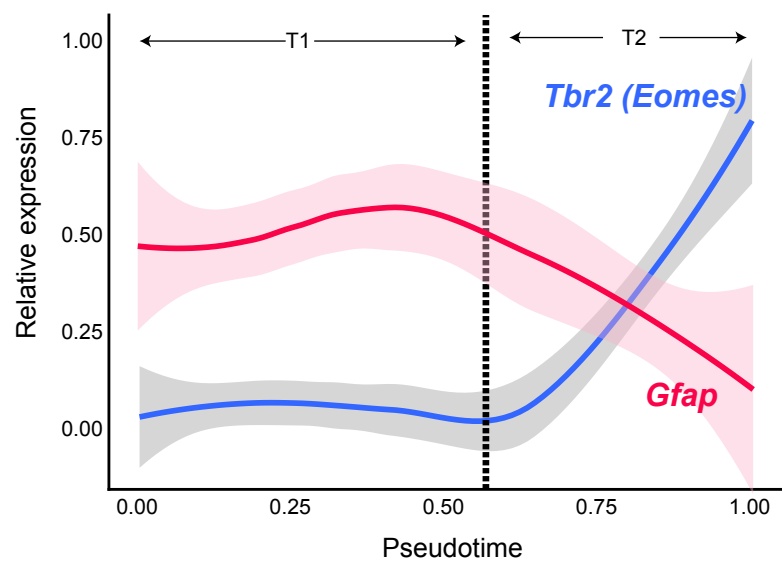

Supplement: S1 Fig — The neurogenic lineage was divided into an early time point (T1) where NSCs express GFAP, and a later time point (T2) where differentiating intermediate neural progenitors begin to express Tbr2. (A) Waterfall plot illustrates the relative expression of Tbr2 and GFAP along a trajectory of adult neurogenesis, as described in Fig 1A. The cutoff point between time points T1 and T2 was determined by the time of increasing Tbr2 expression (last stationary point of the Tbr2 regression line). Data used to generate this figure can be found in the Supporting information Excel spreadsheet (S1 Data). GFAP, glial fibrillary acidic protein; NSC, neural stem cell; Tbr2, T-box brain protein 2. (PDF) [file pbio.3001311.s001.pdf]

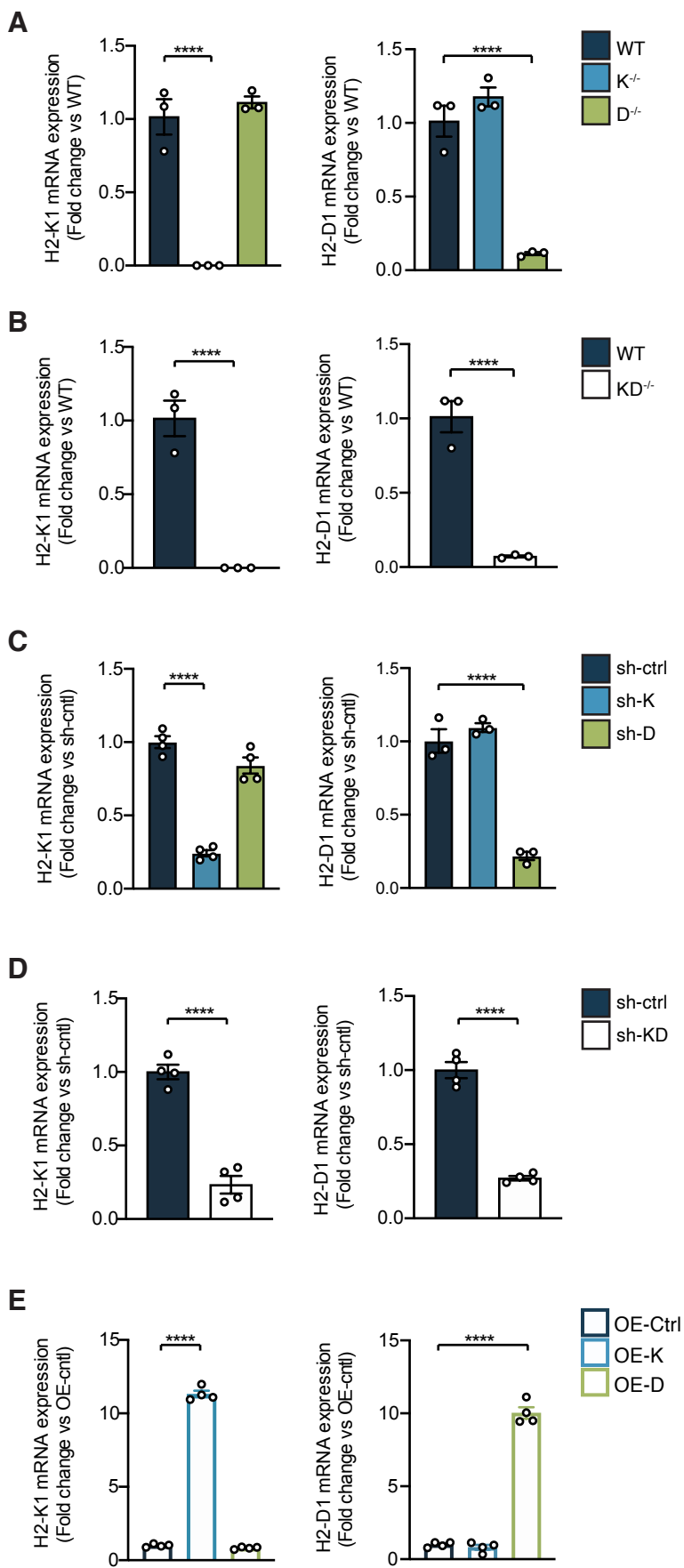

Supplement: S2 Fig — Quantitative RT-PCR of H2-Kb (left panel) and H2-Db (right panel) mRNA from primary NSPCs isolated from WT, K−/−, and D−/− knockout mice (A). n = 3 replicates/group. Quantitative RT-PCR of H2-Kb (left panel) and H2-Db (right panel) mRNA from primary NSPCs isolated from WT and KD−/− double knockout mice (B). n = 3 replicates/group. Quantitative RT-PCR of H2-Kb (left panel) and H2-Db (right panel) mRNA from primary WT NSPCs infected with lentiviruses encoding shRNA targeting H2-Kb (sh-K) or H2-Db (sh-D) or luciferase control (sh-Ctrl) 72 hours after infection (C). n = 4 replicates/group. Quantitative RT-PCR of H2-Kb (left panel) and H2-Db (right panel) mRNA from primary WT NSPCs infected with lentiviruses encoding shRNA concomitantly targeting both H2-Kb and H2-Db (sh-KD) or luciferase as a control (sh-Ctrl) (D). n = 4 replicates per group. Quantitative RT-PCR of H2-Kb (left panel) and H2-Db (right panel) mRNA from primary WT NSPCs infected with lentiviruses overexpressing H2-Kb (OE-K), H2-Db (OE-D), or GFP as a control (OE-ctrl) under the Nestin promoter (C). n = 4 replicates/group. All data represented as mean ± SEM; one-way ANOVA with Dunnett’s post hoc test (A, C, E) and Student t test (B,D); ****p < 0.0001. Data used to generate this figure can be found in the Supporting information Excel spreadsheet (S1 Data). MHC I, major histocompatibility complex class I; NSPC, neural stem and progenitor cell; RT-PCR, reverse transcription polymerase chain reaction; shRNA, short hairpin RNA; WT, wild-type. (PDF) [file pbio.3001311.s002.pdf]

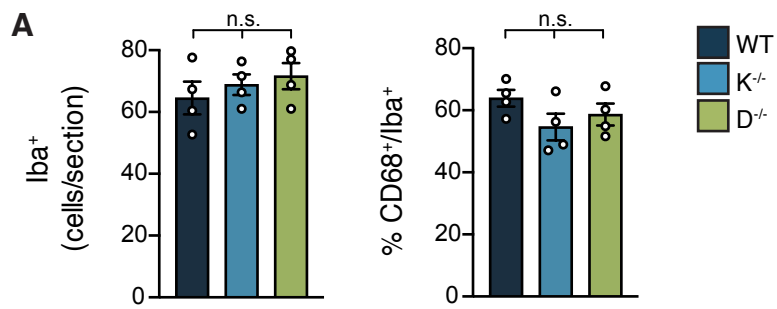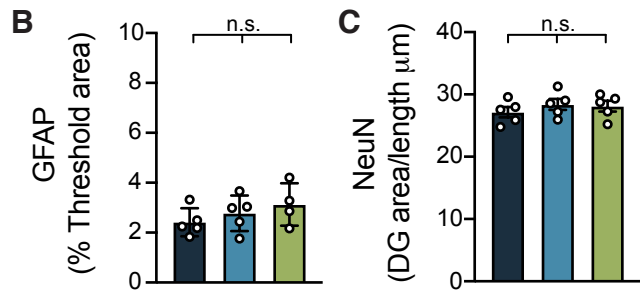

Supplement: S3 Fig — (A) Quantification of Iba1 (a microglial marker) and the percent of microglia coexpressing the activation marker CD68 in the DG of 2–3 months old WT, K−/−, and D−/− knockout mice. n = 4 animals/group (3–6 sections/animal). (B, C) Quantification of GFAP-positive astrocytes (B) or overall DG size using the neuronal marker NeuN (C). n = 4–5 animals/group (3–4 sections/animal). All data represented as mean ± SEM. ANOVA with Dunnett’s post hoc test. Data used to generate this figure can be found in the Supporting information Excel spreadsheet (S1 Data). CD68, cluster of differentiation 68; DG, dentate gyrus; GFAP, glial fibrillary acidic protein; Iba1, ionized calcium-binding adapter molecule 1; MHC I, major histocompatibility complex class I; NeuN, neuronal nuclei; n.s., not significant; WT, wild-type. (PDF) [file pbio.3001311.s003.pdf]

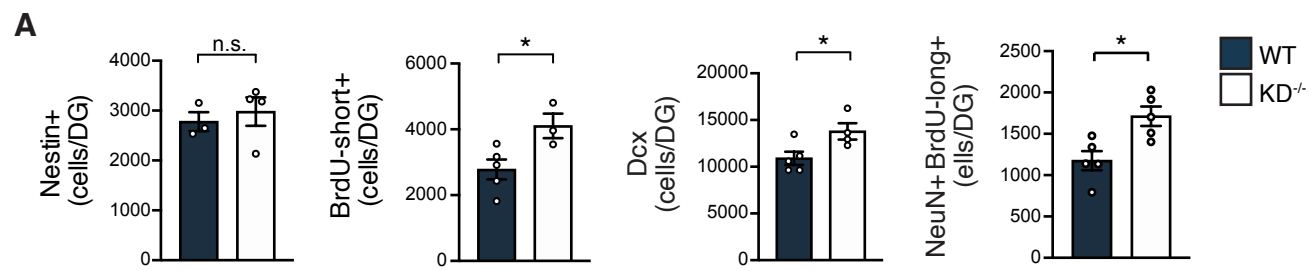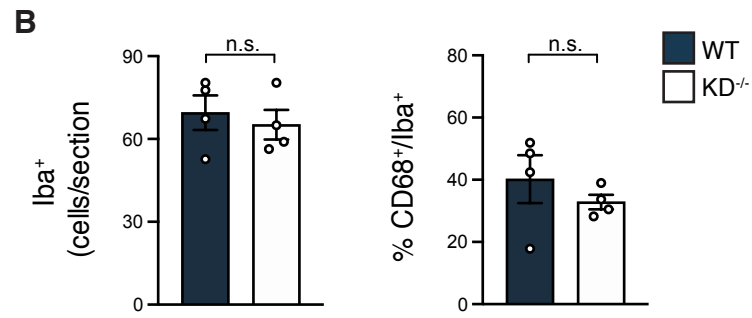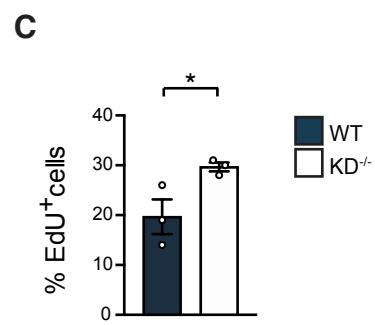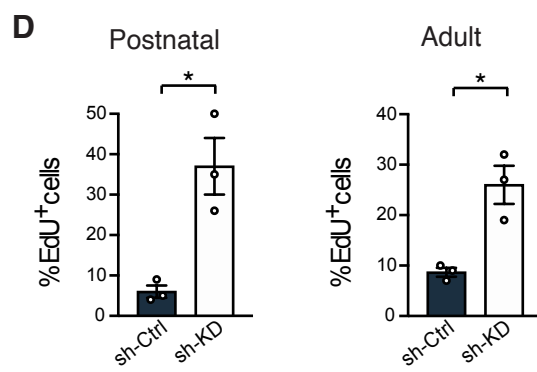

Supplement: S4 Fig — (A) Neurogenesis was characterized in 2-month-old WT and H2-Kb/H2-Db double-knockout (KD−/−) mice. Quantifications of Nestin+ NSCs, BrdU+ short-term proliferating cells, Dcx+ neuroblasts, and adult-born neurons coexpressing BrdU-long and NeuN in the DG. n = 3–5 animals/group (3–6 hippocampal sections/animal). (B) Quantification of Iba+ microglia and the percent of Iba+ microglia expressing CD68 in the DG of adult (2 months) WT or KD−/− mice. N = 4 animals/group (3–6 hippocampal sections/animal). (C) Primary NSPCs isolated from the hippocampi of WT, KD−/− mice were cultured under self-renewal conditions and treated with EdU for 6 hours. Percentage of EdU+ cells are shown (C). n = 3 replicates/group. (D) WT postnatal (left panel) and adult (right panel) NSPCs infected with lentiviruses encoding sHRNA concomitantly targeting H2-Kb and H2-Db (sh-KD) or targeting luciferase as a control (sh-Ctrl) were cultured under self-renewal conditions and treated with EdU for 6 hours. Percentage of EdU+ cells are shown (D). n = 3 replicates/group. All data represented as mean ± SEM. Student t test; *p < 0.05. Data used to generate this figure can be found in the Supporting information Excel spreadsheet (S1 Data). BrdU, 5-bromo-2′-deoxyuridine; CD68, cluster of differentiation 68; Dcx, Doublecortin; DG, dentate gyrus; EdU, 5-ethynyl-2′-deoxyuridine; Iba1, ionized calcium-binding adapter molecule 1; NeuN, neuronal nuclei; n.s., not significant; NSC, neural stem cell; NSPC, neural stem and progenitor cell; shRNA, short hairpin RNA; WT, wild-type. (PDF) [file pbio.3001311.s004.pdf]

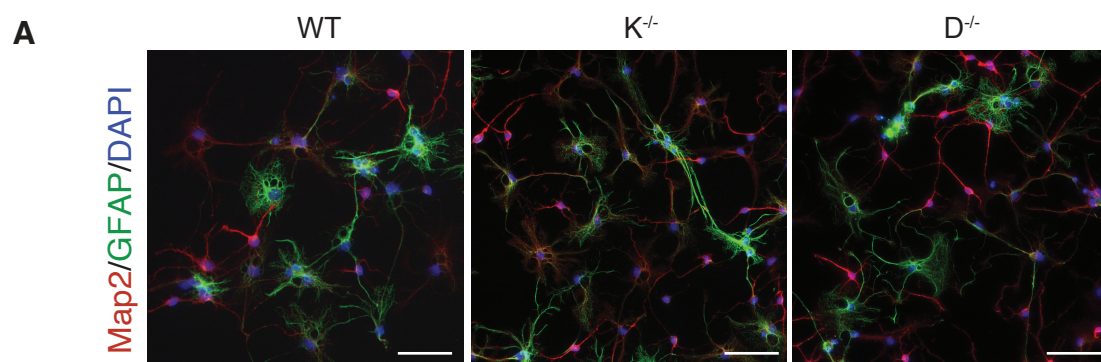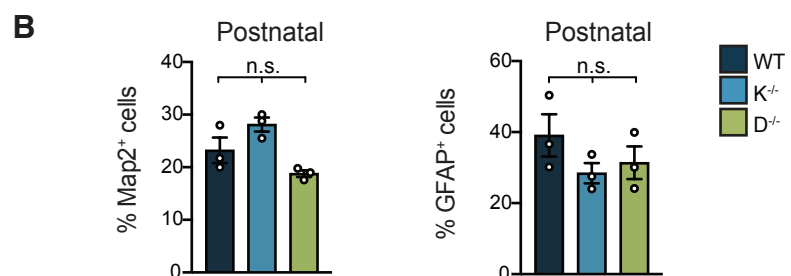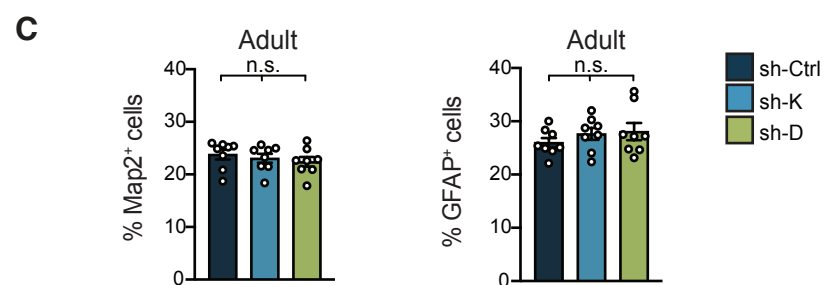

Supplement: S5 Fig — WT, K−/−, and D−/− NSPCs were cultured under growth factor-free conditions and allowed to differentiate for 6 days. Representative field (A) and quantification (B) of Map2+ neurons and GFAP+ astrocytes. Data represented as mean percentages ± SEM; n = 3 replicates per group; ANOVA, with Dunnett’s post hoc test (B). (C) WT adult NSPCs were infected with shRNA lentiviruses against H2-Kb (sh-K), H2-Db (sh-D), or luciferase (sh-Ctrl). Seventy-two hours after infection, NSPCs were transitioned to growth factor-free culturing conditions and allowed to differentiate for 6 days. Quantification (C) of Map2+ neurons (left panel) and GFAP+ astrocytes (right panel). All data represented as mean percentages ± SEM; n = 8 replicates/group. ANOVA with Dunnett’s post hoc test. Data used to generate this figure can be found in the Supporting information Excel spreadsheet (S1 Data). GFAP+, glial fibrillary acidic protein-positive; Map2+, microtubule-associated protein 2-positive; n.s., not significant; NSPC, neural stem and progenitor cell; shRNA, short hairpin RNA; WT, wild-type. (PDF) [file pbio.3001311.s005.pdf]

**A**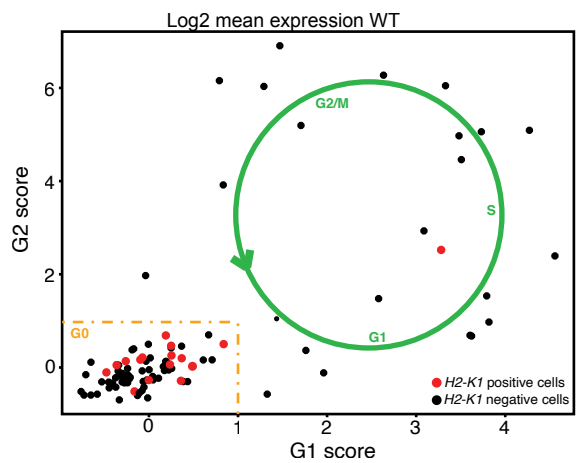**B**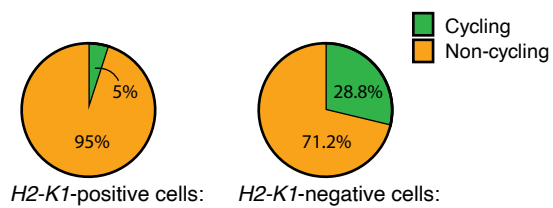**C**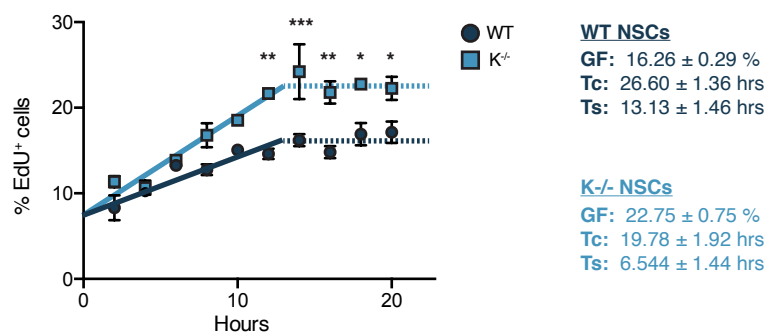

Supplement: S6 Fig — (A, B) A publicly available dataset of single adult hippocampal NSCs [27] was sorted by expression of G1 (x-axis) and G2 (y-axis) cell cycle scores as described in Tirosh and colleagues [29]. Cells positive for H2-K1 expression are indicated in red (A). (B) The percentage of cycling cells was found to be lower in H2-K1-positive cells (5.6%, 1 cycling cell, 17 noncycling cells) versus H2-K1-negative cells (24.4%, 20 cycling cells, 62 noncycling cells); odds ratio: 0.18. (C) Primary hippocampal WT and K−/− NSPCs cultured under self-renewal conditions were treated with EdU at 2, 4, 6, 8, 10, 12, 14, 16, 18, 20, 22, or 24 hours prior to fixation, and percentage of EdU+ cells was determined by flow cytometry. Percent of cells incorporating EdU reached a plateau at 13.47 hours for WT NSPCs and 13.24 hours for K−/− NSPCs (Tc − Ts) when the cycling population (GF) entered the S phase. Linear regression for increasing phase of WT NSPCs: y = 0.6133 × + 7.996; R2 = 0.829 and K−/− NSPCs: y = 1.165 × + 7.406; R2 = 0.889. Values for cell cycle parameters calculated from cumulative labeling are shown in the right panel. (C). n = 3 replicates per group for in vitro experiments. Data represented as mean ± SEM; two-way repeated measures ANOVA with Sidak’s post hoc test (K); *p < 0.05, **p < 0.01, ***p < 0.001. Data used to generate this figure can be found in the Supporting information Excel spreadsheet (S1 Data). EdU, 5-ethynyl-2′-deoxyuridine; GF, growth fraction; NSC, neural stem cell; NSPC, neural stem and progenitor cell; Tc, length of cell cycle; Ts, length of S phase; WT, wild-type. (PDF) [file pbio.3001311.s006.pdf]

**A**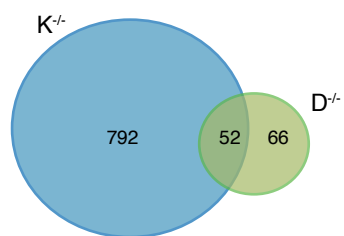**B**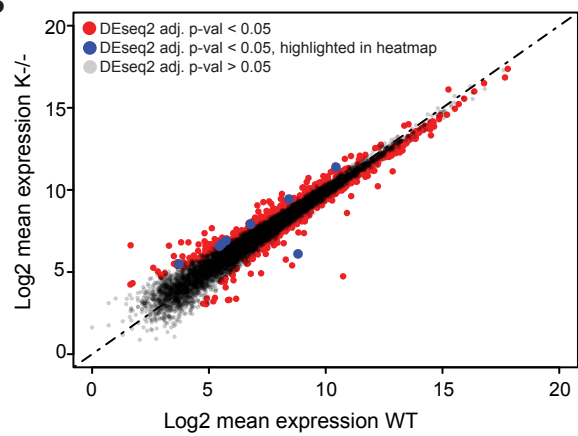

Supplement: S7 Fig — RNA-seq analysis of primary hippocampal WT, K−/−, and D−/− NSPCs cultured under self-renewal conditions. (A) Venn diagram illustrating the number of genes differentially expressed (DESeq2 adj. p < 0.05) between WT, K−/−, and D−/− NSPCs. (B) Scatterplot of the average gene expression in WT (x-axis) and K−/− (y-axis) NSPCs. Differentially expressed genes (DESeq2 adj. p < 0.05, 432 down- and 408 up-regulated) are indicated in red. Genes shown in heatmap (Fig 3B) are colored in blue. Data used to generate this figure can be found in the Supporting information Excel spreadsheet (S1 Data). NSPC, neural stem and progenitor cell; RNA-seq, RNA-sequencing; WT, wild-type. (PDF) [file pbio.3001311.s007.pdf]

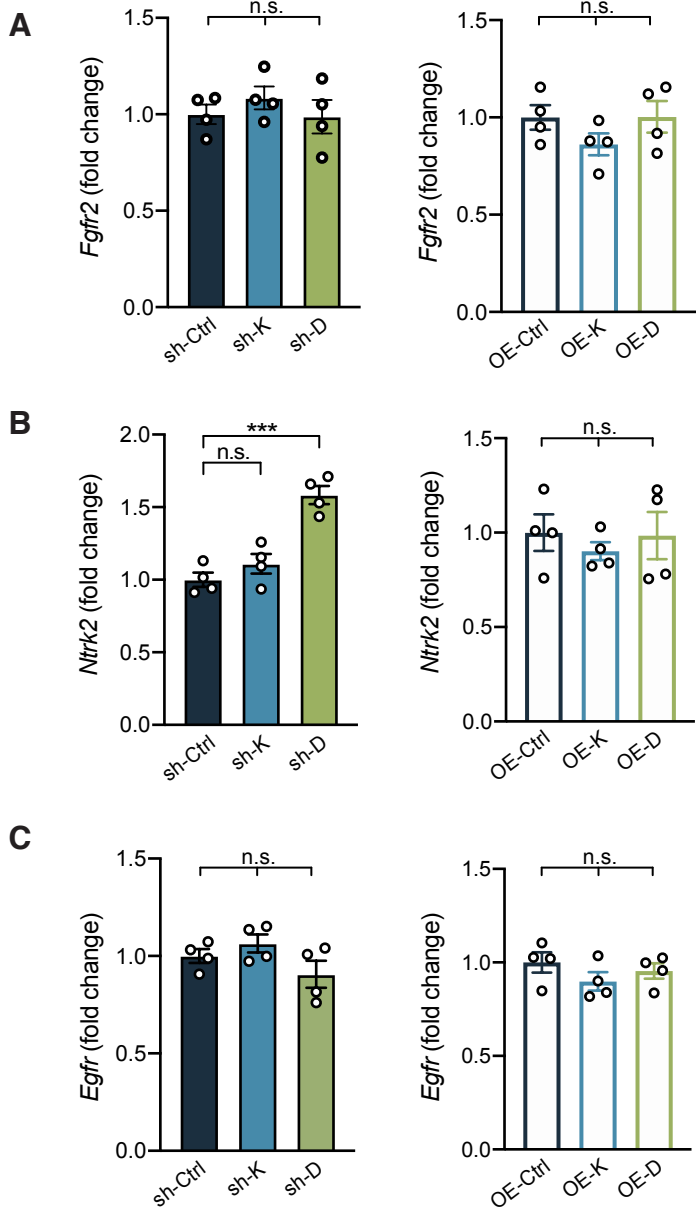

Supplement: S8 Fig — WT adult NSPCs were infected with either sh-K, sh-D, and sh-control (sh-Ctrl) or Nestin-driven H2-Kb (OE-K), H2-Db (OE-D), and GFP control (OE-Ctrl) overexpression lentiviruses. NSPCs were grown under self-renewal conditions, and Fgfr2 (A), Ntrk2 (B), and Egfr (C) expression was measured using quantitative RT-PCR 72 hours after infection. n = 4 replicates/group. All data represented as mean ± SEM; one-way ANOVA with Dunnett’s multiple comparisons test; *p < 0.05; ***p < 0.001. Data used to generate this figure can be found in the Supporting information Excel spreadsheet (S1 Data). Egfr, epidermal growth factor receptor; Fgfr2, fibroblast growth factor receptor 2; GFP, green fluorescent protein; n.s., not significant; NSPC, neural stem and progenitor cell; Ntrk2, neurotrophic tyrosine kinase receptor type 2; RT-PCR, reverse transcription polymerase chain reaction; WT, wild-type. (PDF) [file pbio.3001311.s008.pdf]

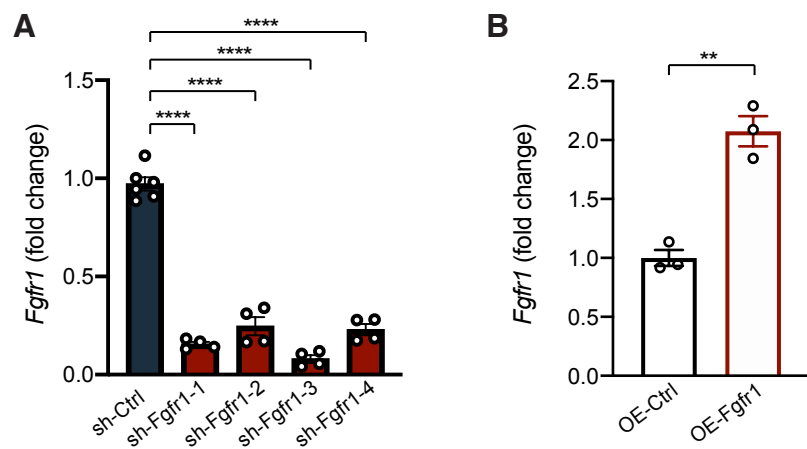

Supplement: S9 Fig — (A) WT adult NSPCs were infected with 4 different shRNAs targeting Fgfr1 (sh-Fgfr1) or sh-control (sh-Ctrl) lentiviruses. NSPCs were grown under self-renewal conditions, and Fgfr1 expression was measured using quantitative RT-PCR 72 hours after infection. sh-Fgfr1(2) was selected for additional experiments. n = 4–5 replicates/group. (B) WT adult NSPCs were infected with a Nestin-driven Fgfr1 (OE-Fgfr1) or GFP control (OE-Ctrl) overexpression lentiviruses. NSPCs were grown under self-renewal conditions, and Fgfr1 expression was measured using quantitative RT-PCR 72 hours after infection. All data represented as mean ± SEM; one-way ANOVA with Dunnett’s multiple comparisons test (A) and Student t test (B); **p < 0.01; ****p < 0.0001. Data used to generate this figure can be found in the Supporting information Excel spreadsheet (S1 Data). Fgfr1, fibroblast growth factor receptor 1; GFP, green fluorescent protein; NSPC, neural stem and progenitor cell; RT-PCR, reverse transcription polymerase chain reaction; shRNA, short hairpin RNA; WT, wild-type. (PDF) [file pbio.3001311.s009.pdf]

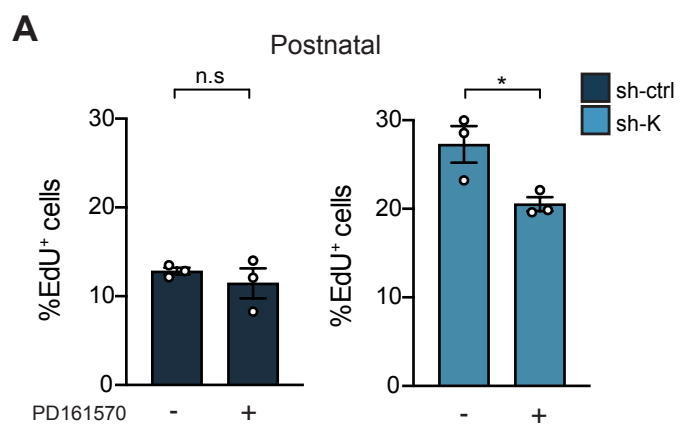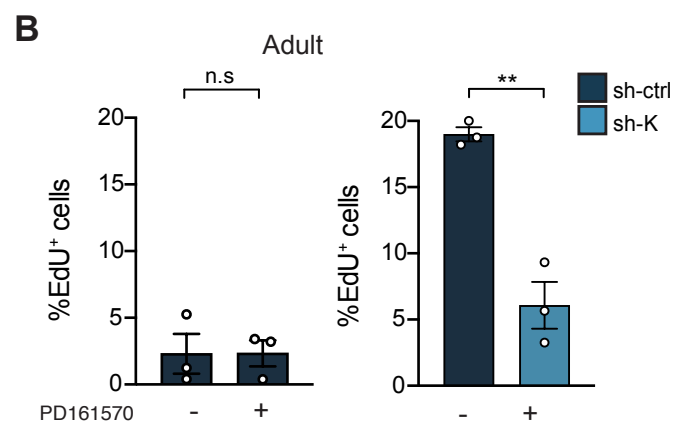

Supplement: S10 Fig — WT postnatal (D) and adult (E) NSPCs were infected with lentiviruses encoding sh-K and sh-control (sh-Ctrl), cultured under self-renewal conditions and exposed to Fgfr inhibitor PD 161570 (PD, 0.1 uM) or phosphate-buffered saline control for 1 hour and subsequently treated with EdU for 6 hours to test for proliferation (D, E). Data represented as mean percentage of EdU+ cells ± SEM. n = 3 replicates/group. Student t test; *p < 0.05 and **p < 0.01. Data used to generate this figure can be found in the Supporting information Excel spreadsheet (S1 Data). EdU, 5-ethynyl-2′-deoxyuridine; Fgfr, fibroblast growth factor receptor; n.s., not significant; NSPC, neural stem and progenitor cell; WT, wild-type. (PDF) [file pbio.3001311.s010.pdf]

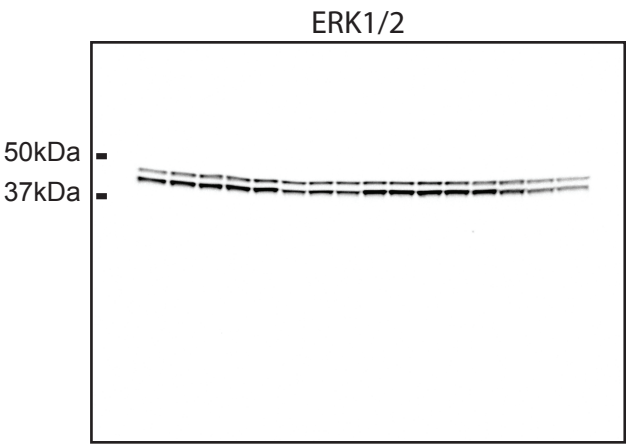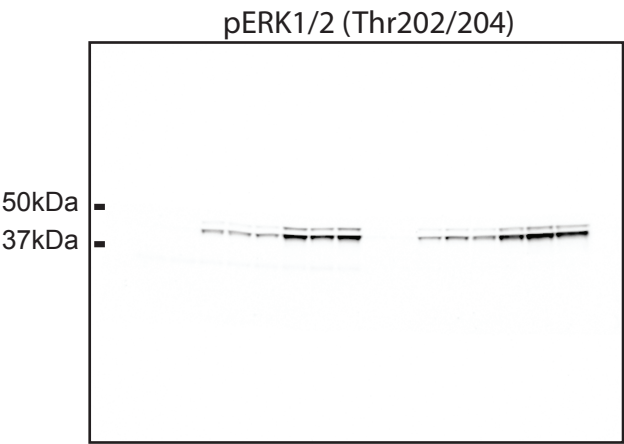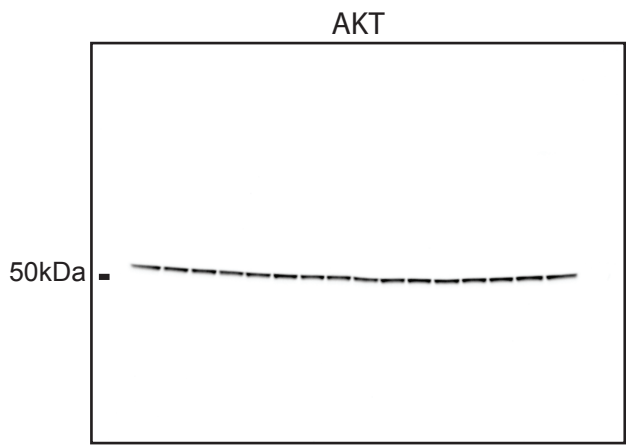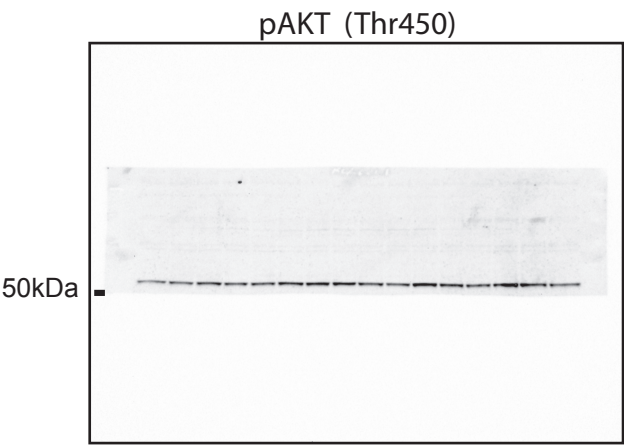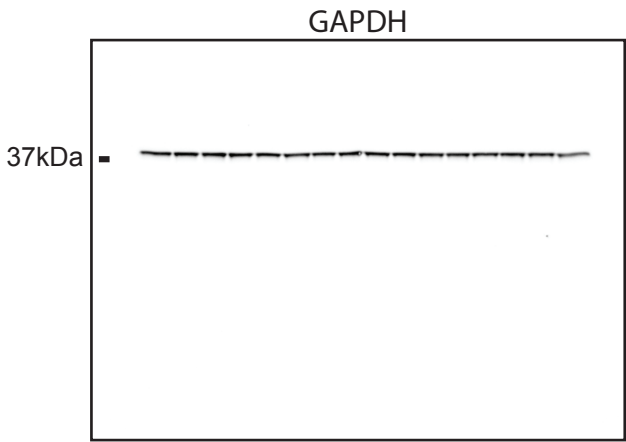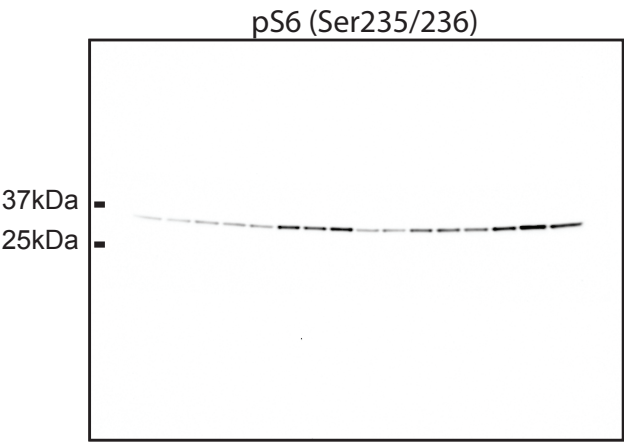

Supplement: S1 Raw Images — (PDF) [file pbio.3001311.s012.pdf]
