# Supplementary material for: MHC class I H2-Kb negatively regulates neural progenitor cell proliferation by inhibiting FGFR signaling
Source: PLoS Biol. 2021 Jun 28;19(6):e3001311. doi: 10.1371/journal.pbio.3001311 (PMC8270425; doi:10.1371/journal.pbio.3001311)
Supplement: S1 Table — (PDF) [file pbio.3001311.s013.pdf]

S1 Table

REAGENT or RESOURCE	SOURCE	IDENTIFIER
**Antibodies**		
Rabbit polyclonal anti-Actin	Sigma-Aldrich	Cat # A5060
Rat monoclonal anti-BrdU	Thermo Fisher Scientific	Cat # MA1-82088
Rat monoclonal anti-CD68	Bio-Rad	Cat # MCA1957
Goat polyclonal anti-Doublecortin	Santa Cruz	Cat # sc-8066
Rabbit polyclonal anti-Erk1/2	Cell Signaling	Cat # 4695
Mouse monoclonal anti-GAPDH	Abcam	Cat # Ab8245
Rabbit polyclonal anti-GFAP	Dako	Cat # Z0334
Rabbit polyclonal anti-Iba1	Wako	Cat # 01919741
Mouse monoclonal anti-Map2	Sigma-Aldrich	Cat # M9942
Mouse monoclonal anti-NeuN	Millipore	Cat # MAB377
Mouse monoclonal anti-Nestin	Millipore	Cat # MAB353
Rabbit polyclonal anti-phospho Akt (Tyr450)	Millipore	Cat # 07-1643
Rabbit monoclonal anti-Phospho-Akt (Thr450)	Cell Signaling	Cat # 12178
Rabbit monoclonal anti-pan Akt	Cell Signaling	Cat # 4691
Rabbit polyclonal anti-phospho Erk1/2 (Thr202/Tyr204)	Cell Signaling	Cat # 4370
Rabbit polyclonal anti-phospho S6 (Ser235/236)	Cell Signaling	Cat # 2211
Rabbit polyclonal anti-TurboGFP	Thermo Fisher Scientific	Cat # PA5-22688
Donkey anti-Rabbit: Alexa-488 conjugated secondary antibody	Thermo Fisher Scientific	Cat # A21206
Donkey anti-Rabbit: Alexa-555 conjugated secondary antibody	Thermo Fisher Scientific	Cat # A3157
Donkey anti-Rat: Alexa-488 conjugated secondary antibody	Jackson ImmunoResearch	Cat # 712-165-150
Donkey anti-Goat: Alexa-555 conjugated secondary antibody	Thermo Fisher Scientific	Cat # A21432
Donkey anti-Mouse: Alexa-488 conjugated secondary antibody	Thermo Fisher Scientific	Cat # A21202
Donkey anti-Mouse: Alexa-555 conjugated secondary antibody	Thermo Fisher Scientific	Cat # A31570
Donkey anti-Mouse: Alexa-647 conjugated secondary antibody	Thermo Fisher Scientific	Cat # A31571
Donkey anti-Rabbit HRP-conjugated secondary antibody	GE Healthcare	Cat # NA934
Goat anti-Guinea Pig HRP-conjugated secondary antibody	Santa Cruz	Cat # sc-2438
Goat anti-Mouse HRP-conjugated secondary antibody	Millipore	Cat # AP124P
**Bacterial and Virus Strains**		
pGreenPuro lentivirus shRNA expression system	System Biosciences	Cat # SI505A-1
**Chemicals, Peptides, and Recombinant Proteins**		
5-bromo-2’-deoxyuridine (BrdU)	Sigma-Aldrich	Cat # B5002
5-ethynyl-2’-deoxyuridine (EdU)	Life Technologies	Cat # E10187
Recombinant Epidermal Growth Factor (EGF)	Peprotech	Cat # AF-100-15

Recombinant Fibroblast Growth Factor-basic (FGF)	Peprotech	Cat # 100-18B
PD 161570 (selective FGFR inhibitor)	Tocris	Cat # 3724
Hoechst 33342	Thermo Fisher Scientific	Cat # H3570
Alexa Fluor 488 Azide	Thermo Fisher Scientific	Cat # A10266
Alexa Fluor 555 Azide	Thermo Fisher Scientific	Cat # A20012
Alexa Fluor 647 Azide	Thermo Fisher Scientific	Cat # A10277
**Critical Commercial Assays**		
Click-iT EdU Alexa Fluor Imaging Kit	Thermo Fisher Scientific	Cat # C10337
Click-iT Plus EdU Alexa Fluor Flow Cytometry Assay Kit	Thermo Fisher Scientific	Cat # C10634
Rneasy Mini Kit	Qiagen	Cat # 74106
High Capacity cDNA Reverse Transcription Kit	Life Technologies	Cat # 4368814
Power SYBR Green PCR Master Mix	Life Technologies	Cat # 4367659
Kapa HiFi HotStart ReadyMix	Kapa Biosystems	Cat # KK2601
Nextera XT DNA Library Prep Kit	Illumina	Cat # FC-131-1096
qPCR Library Quantification Kit	Kapa Biosystems	Cat # KK 4824
**Deposited Data**		
Single cell sequencing data	Shin et al., 2015	GEO: GSE71485
Raw data files for RNA sequencing: NSCs	Current Manuscript	ArrayExpress: E-MTAB-5827
**Experimental Models: Organisms/Strains**		
Mouse: C57BL/6Ntac	Taconic	Model # B6-M
Mouse: B6.129P2-*H2-Kb*^{tm1} *H2-Db*^{tm1} N12	Taconic	Model # 4215-M
Mouse: B6.129P2-*H2-Kb*^{tm1} N12	Taconic	Model # 4216-M
Mouse: B6.129P2-H2-*Db*^{tm1} N12	Taconic	Model # 4217-M
Mouse: C57BL/6J	Jackson	Model # 000664
**Recombinant DNA and Oligonucleotides**		
pGreenPuro shRNA Cloning and Expression Lentivector	System Biosciences	Cat # SI505A-1
Quantitative real-time PCR primers	Life Technologies	Sequences in Method details
shRNA primers	Life Technologies	Sequences in Method details
Nestin H2-K1/H2-D1/GFP/Fgfr1 expression construct	Current Manuscript	Method details
**Software and Algorithms**		
GraphPad Prism 6 and 8	GraphPad	6 and 8
Trim Galore! (https://www.bioinformatics.babraham.ac.uk/projects/trim_galore/)	Babraham Bioinformatics	0.4.1

HISAT2 (https://ccb.jhu.edu/software/hisat2/index.shtml)	Center for Computational Biology, Johns Hopkins University	2.0.4
Subread FeatureCounts (http://bioinf.wehi.edu.au/featureCounts/)	Walter + Eliza Hall Bioinformatics	1.5.0
R (https://cran.r-project.org/)	Comprehensive R Archive Network	3.2.5
DESeq2 BioC (https://bioconductor.org/packages/release/bioc/html/DESeq2.html)	Bioconductor	3.2
ConsensusPathDB – Mouse (http://cpdb.molgen.mpg.de/MCPDB)	Max-Planck-Institute for Molecular Genetics	Release MM9
ImageJ/FIJI	NIH	1.8.0_91 and 2.1.0

CONTACT FOR REAGENT AND RESOURCE SHARING

Please contact Saul Villeda (saul.villeda@ucsf.edu) for further information and requests for resources and reagents generated in this study.
