# Supplementary material for: MHC class I H2-Kb negatively regulates neural progenitor cell proliferation by inhibiting FGFR signaling
Source: PLoS Biol. 2021 Jun 28;19(6):e3001311. doi: 10.1371/journal.pbio.3001311 (PMC8270425; doi:10.1371/journal.pbio.3001311)
Supplement: Attachment — Submitted filename: Lin_PLOSBIO_Response-to-Reviewers_041521.pdf [file pbio.3001311.s014.pdf]

Response to reviewer comments of manuscript “MHC Class I H2-K^b Negatively Regulates Neural Progenitor Cell Proliferation by Inhibiting FGFR Signaling” (PBIOLOGY-D-20-02289R1)

We would like to thank the reviewers for their helpful and constructive comments, which we have taken to heart and have accordingly performed a substantial number of new experiments. The reviewers’ comments have helped us to improve the quality of our study and the results of these new experiments provide a more complete understanding of how the MHC Class 1 molecule, H2-K^b, regulates neural stem and progenitor cell (NSPC) proliferation. We would like to note that due to restrictions in place as a result of the Covid-19 pandemic no new genetic knockout mouse models could be generated; however, all new loss and gain of function experiments were performed using viral-mediated approaches in adult wild-type mice. In particular, using *in vitro* and *in vivo* viral-mediated RNA interference (RNAi) and overexpression approaches we tease apart a role for H2-K^b, and not H2-D^b, in regulating adult NSCP proliferation and adult hippocampal neurogenesis, and provide further mechanistic evidence for the involvement of Fgfr1 signaling in this process. We hope the revised version of our manuscript satisfactorily addresses the issues that were raised.

Please find below a point-by-point discussion to the reviewers’ comments. In our reply, the questions/comments are in black and our replies are in blue font.

Reviewer #1: In their work Li et al. investigate the MHC molecule H2K1 as a regulator of proliferation during hippocampal neurogenesis. Using a mix of mouse genetics, in vitro analyses, expression analyses and biochemistry they provide evidence that H2K1 acts as a negative regulator of DG neurogenesis, acting not directly on the stem cells, but at later stages. They also claim the this action is mediated by the FGFR pathway.

The paper is well written. The mouse genetics approach is well designed and the analyses of these animals are convincing. The use of RNAi represents a good support for the mouse mutation analyses. Overall this is an interesting paper that has potential for publication in PLOSBio. However, there are some issues, in particular in the last FGFR-related part of the manuscript, that dampen my enthusiasm and that should be addressed.

First, as said, the loss of function studies in Fig. 1 are well performed and demonstrate that H2K1 is necessary to dampen DG neurogenesis. It would be interesting, and nicely complementary, to see what happens if the molecule is overexpressed and/or maintained at later stages in stem cells, in a gain of function approach. Is it sufficient to decrease proliferation? Is downregulation of H2K1 necessary? As the authors use the lentiviral approach for the RNAi study, this should not be too difficult.

We thank the Reviewer for their positive and constructive feed-back. To address the Reviewer’s comments, we generated novel lentiviral constructs encoding either H2-K1, H2-D1 or GFP control driven by the mouse Nestin promoter and overexpressed them in adult NSPCs *in vitro* and *in vivo*. In summary, overexpression of H2-K1, but not H2-D1, significantly decreases proliferation of adult NSPCs *in vitro* and reduces the number of short-term proliferating NPCs, DCX-positive neuroblasts and newly incorporated neurons in the adult hippocampal neurogenic niche *in vivo*. These new data are now included in the new Figure 2A-E as well as Figure S2E and S5C.

Second, the expression, biochemical and pharmacological data in Fig. 3 appears fragmentary

and unclear. The expression analysis shows that several GF receptors (FRGG1-3 /EGFR/Ntrk2) are expressed in NPSCs, but only FGFR1 is then presented in detail in the mutant contexts (3b). This data would be far more convincing with a systematic analysis of the expression of all GFRs in the mutants. Moreover, if only FGFR is a candidate, why is there EGF in the GF-mix in Fig. 3C. The pharmacological approach is too limited. Again, a systematic and complete analysis using several pharmacological interventions would be necessary to demonstrate specificity of the FGFR pathway. To my eyes this entire part is rather correlative and indirect.

We now systematically characterize the growth factor receptor expression of Fgfr1, Fgfr2, Fgfr3 Egfr and Ntrk2 in adult NSPCs following viral-mediated abrogation and overexpression of H2-K1 or H2-D1 by qPCR (Figure 3E and Figure S8). We did not detect significant level of Fgfr4, Pdgfra, Pdgfrb and Ntrk1. We observed a selective bidirectional change in Fgfr1 and Fgfr3 in adult NSPCs infected with lentivirus encoding shRNA targeting H2-K1 or overexpressing H2-K1, in which Fgfr1 and Fgfr3 expression increased following H2-K1 abrogation and decreased following H2-K1 overexpression (Figure 3E).

Given that Fgfr1 showed highest growth factor receptor expression in WT NSPCs (Figure 3D), and exhibited bidirectional gene expression changes following H2-K^b abrogation and overexpression (Figure 3E), we opted to investigate the functional role of Fgfr1 in mediating the effects of decreased H2-K^b expression on cell proliferation. To address the reviewer's comments with regards to biochemical studies, we repeated the referenced Western blot experiment (previous figure 3C) using adult NSPCs with varying level of FGF in the absence of EGF in the culture media following viral-mediated H2-K1 abrogation (Figure 3F). Additionally, to complement the pharmacological approach used in the previous version of the manuscript, we performed necessity and sufficiency experiments using viral-mediated genetic manipulations of Fgfr1. To this end, we generated novel lentiviral constructs encoding shRNA targeting Fgfr1 or overexpressing Fgfr1 under the control of the mouse Nestin promoter. We now demonstrate that decreasing Fgfr1 expression mitigates the effect of H2-K1 abrogation on NSPC proliferation (Figure 3G). Conversely, mimicking the increase in Fgfr1 expression observed following H2-K^b abrogation by viral-mediated Fgfr1 overexpression increased NSCP proliferation (Figure 3H). We are confident these new data provide a strong link between H2-K1 and Fgfr1 expression.

Reviewer #2: In present study, Lin and co-authors studied the roles of major histocompatibility complex class I (MHC I) molecules, H2-K and H2-D in regulation of neural stem/progenitor cells and adult hippocampal neurogenesis. Specifically, they identified that H2-K, but not H2-D, governed adult neurogenesis by regulating cell cycle dynamics. Using hippocampal NSPCs culture combined with RNA-sequencing, they found that H2-K regulate cell proliferation through inhibiting FGFR-mediated signaling pathway. In general, it is a potential interesting study in the field. But there are still some major concerns that require further attention.

1) The in vivo neurogenesis has been performed on H2-K KO mice. However, the authors have not assessed whether H2-K mutation has developmental consequences on the DG cellular composition, which is highly possible if H2-K is a major actor in neurogenesis. In addition, H2-K deficiency led to increased proliferation of postnatal-derived NSPCs in vitro (Fig. 2B). Therefore, the defective adult hippocampal neurogenesis in H2-K KO mice could be a consequence of developmental defect, but is not specific in adult brain.

The Reviewer raises an interesting point. We performed additional neuronal and glial characterization of the dentate gyrus region of H2-K1 and H2-D1 knockout mice. We do not observe any difference in the dentate gyrus area occupied by neurons and glia using the markers NeuN, GFAP, Iba1 and CD68 (Figure S3). Additionally, we would like to highlight that the shRNA

mediated knockdown of H2-K1 and H2-D1 in the hippocampal neurogenic niche *in vivo* was performed in adult wildtype mice, allowing us to investigate their role in a temporally controlled manner post development (Figure 1E and 1F). The effect of H2-K1 abrogation is consistent with the effect observed in whole-body knockout mice. Similarly, shRNA mediated knock of H2-K1 in wildtype NSPCs yields comparable results to NSPCs derived from H2-K1 knockout animals (Figure 1H, Figure S5A-C). While our data does not exclude a developmental effect of H2-K1 and H2-D1, it does highlight that its role extends to adult NSPCs.

2) Although the bioinformatics analysis showed that the expression of H2-K was expressed in NSPCs and suggested its potential role in regulating adult hippocampal neurogenesis, it is still lacking of the in vivo expression pattern of H2K in adult hippocampal neurogenic niche to argue the specific role of H2K in NSPCs.

In an attempt to address this question, we worked with specialists at ACD Bio to generate H2-K1 and H2-D1-specific RNAScope probes. Unfortunately, we were unable to generate probes since their design requires 300-1000nt long stretches of mRNA with low homology. MHC I molecules, in particular H2-K1/D1, display a high degree of homology and are thus not amenable to this technology. We were able to design and manufacture H2-K1 and H2-D1 BaseScope probes which rely on smaller stretches of homology. While the H2-D1 probe appears to label neurons and microglia in the adult brain, the H2-K1 probe does not pick up any specific signal and displayed high background staining in H2-K1 knockout tissue. Both BaseScope probes are now available to other researchers through ACD Bio (Cat# 714231 and 714221). Additionally, we worked with Covance to generate a guinea pig anti-H2-K^b antibody; however, this antibody was not amenable to immunohistochemistry. Similar results were also obtained using a rabbit anti-H2-K^b antibody that we obtained as a generous gift from Dr. Carla Shatz at Stanford University. While we are unable to provide a characterization of the *in vivo* expression, we believe the newly added rescue experiments in adult NSPCs in which the effects of abrogating endogenous H2-K1 on proliferation could be rescued by overexpressing H2-K^b under the control of a mouse Nestin promoter (Figure 2E) indicate that adult neural stem cell-specific expression of H2-K1 plays a role in negatively regulating proliferation.

3) In adult K-/- mice, more generated new-born neurons (DCX+ cells) are observed, while there is an increased proliferation (Edu+ cells) in cultured K-/- NPSC, which suggested that the increased neurogenesis might be resulted by enhanced proliferation. It is necessary to perform short-term Edu/Brdu or use Ki67/MCM2 combined with cell type specific markers to evaluate proliferative status in vivo.

We agree with the reviewer that the transcriptional and histological analyses are pointing at enhanced proliferation leading to increased neurogenesis in the absence of H2-K1. Thus, we assessed short-term Edu labeling in the adult K-/- mice, adult wild-type mice infected with lentivirus encoding shRNA targeting H2-K1, and adult wild-type mice infected with lentivirus encoding Nestin-driven H2-K1 (Figure 1E-G and Figure 2A-C). Collectively, these data indicate that proliferation is increased following H2-K1 abrogation and decreased following H2-K1 overexpression.

4) Both KD-/- and K-/- mice displayed increased neurogenesis in the adult hippocampus (Fig. 1 D and Fig. 1E, DCX and NeuN). However, it seems the number of Nestin+ cells in KD-/- mice is higher than that in K-/- mice (~3000 Nestin+ cells KD-/- mice [Fig.1E], while ~2000 Nestin+ cells K-/- mice [Fig. 1D]). Further comparison the number of Nestin+ cells between K-/- and KD-/- should be done to conclude whether the defective neurogenesis is due to altering Nestin+ cells in these two mutations.

Since the H2-K1 and H2-D1 genes are located in relatively close-proximity on mouse chromosome 17, the KD-/- double-knock out mutations are co-inherited. Thus, the KD double knock-out line is maintained separately from the single knock out mouse lines. Since neurogenesis is sensitive to age (let alone a series of systemic and environmental manipulations), all histological analysis of the K-/-, D-/- and KD-/- mice were always performed on age and gender-matched littermate controls. Within the lab, we generally do not compare neural stem cell numbers between animals from different cohorts since they can easily be impacted by relatively small differences in age, experimenter and analysis methods. We would like to note that the K-/- and D-/- mice and their age-matched controls were 3 months of age and the KD-/- mice and their age-matched controls were 2 months of age. While we are unable to directly compare the K-/- to the KD-/- mice, the number of Nestin+ cells did not significantly differ between the K-/- mice and their age-matched controls and the KD-/- mice and their age-matched control group (Figure 1D and Supplementary figure 4A). Due to the substantial additional data included in the revised manuscript, we now provide K-/- and D-/- data in Figure 1 and KD-/- data in Figure S4 with the age of all genotypes clearly stated in the corresponding figure legends.

Reviewer #3: In manuscript PBIOLOGY-D-20-02289, Lin et al. address the important issue of whether MHCI molecules regulate neural stem cell proliferation and neurogenesis. The authors first performed transcriptomic analyses of Neural Stem and Progenitors Cells (NSPCs) by analyzing a publicly available dataset of Nestin positive cells and their immediate progeny. Through this analysis, Lin et al found that the class I MHC H2-Kb was downregulated in NSCs that were more neurogenic in their transcriptomic signature, while H2-Db did not change significantly. The authors follow up with several of loss of function experiments with knockout and knockdown of the 2 Class I MHC's (H2Kb, H2Db) both separately and together, that implicate H2Kb in repressing the proliferation of NPSCs. This change in proliferation is described as an increase in DCX + and NeuN/Brdu+ cells in the DG of the adult hippocampus. In vitro experiments using primary NPSCs from both postnatal and adult mice showed that the % of EDU+ cells increases significantly when H2K is knocked out or knocked down with RNAi. Finally, transcriptomic analysis of NSPCs from H2K-/- mice showed a large difference in the transcriptomic signature compared to WT and H2D-/- cells. Specifically, cells that have elevated levels of H2K are less likely to have a cycling (proliferative) transcriptomic signature and FGFR1 mRNA is upregulated in K-/- NSPCs. They also found that an FGFR pharmacological inhibitor reduces the large increase in the % of Edu+ proliferating cells caused by H2K knoc down. The authors conclude that MHC I is required to regulate NSPC proliferation through inhibiting FGFR1 signaling.

Although these data are novel and address an important gap in our knowledge, there are several concerns that need to be addressed.

(1) The Western blot experiments should have been performed in the presence of phosphatase inhibitors to preserve the phosphorylation status of these proteins after lysis. There is also no quantification displayed, which is critical to assess any significant differences after normalization of pERK to ERK and ERK and other proteins to GAPDH or Actin. Finally, the authors should indicate how many repeats of this biochemical experiment were performed; they are typically perfoemed in triplicate for quantification.

We thank the reviewer for the feed-back and for identifying an important lack of detail in the method section. We agree that the detection of phospho-proteins is sensitive to dephosphorylation in the absence of inhibitors. Thus, all cells were routinely lysed on ice in RIPA

buffer in the presence of both proteinase inhibitor and phosphatase inhibitor cocktails. The Western blot method section was adapted accordingly. Additionally, we repeated the signaling analysis in adult NSPCs following viral-mediated abrogation of H2-K1 and included the new representative Western blots with quantifications in the revised manuscript (Figure 3F).

(2) It is unclear if the effects of MHCI on proliferation are truly specific and/or limited to H2K? Given that there is residual H2D mRNA expression in the H2D knockout, and the nominally higher levels of proliferative cells in the KD-/-, would it be more accurate to say that there remains a possibility of H2D having a role (although limited) in NSPC proliferation? One experiment to address this could be to express H2K and H2D In knockout K-/- KD-/- primary NSPCs, expecting to see a reduction in proliferation. Can H2D replace H2K to inhibit proliferation in this context?

The H2-D1 knockout mouse is lacking the first three exons which includes approximately half of its coding sequence, including the N-terminal signal peptide for proper membrane insertion and trafficking. However, as the reviewer indicates, the residual mRNA can still be transcribed and detected by qPCR using sets of primers that are in the unaffected 3' region of the mRNA. While we cannot exclude that shorter peptides may still be generated, the H2-D1 knockout mice do not generate a full-length protein.

In line with the Reviewer's suggestion, we examined whether the effects of abrogating H2-K^b on NSPC proliferation could be rescued by overexpressing either H2-K^b or H2-D^b. Adult NSPCs were infected with lentivirus encoding shRNA targeting H2-K1 in combination with lentivirus encoding H2-K1 or H2-D1 under the control of the mouse Nestin promoter. Consistent with our previous findings, abrogation of H2-K^b resulted in an increase in EdU-positive proliferating cells (Figure 2E); however, this effect was mitigated by overexpression of H2-K^b but not H2-D^b (Figure 2E). These data indicate that functionally, H2-D1 is not able to compensate for the lack of H2-K1 in NPCs. We thank the reviewer for the suggestion.

(3) Data should be plotted by dot plot rather than bar graphs so that the variability can be more easily assessed.

All bar graphs were updated accordingly.

(4) Figure 1: Panels D and E. The values for WT Dcx+ cells (~5K vs. ~10 K) and NeuN+BrDU+ cells (~600 vs. ~1200) are very different between the 2 experiments, whereas the control numbers of nestin+ cells are similar (~28K in both experiments). This raises the question of whether the WT used for each comparison are the appropriate controls, or whether the WT values are driving the result. The authors should clarify if the WT mice used for the comparisons to the single KO lines were littermates or a separate line and if the latter, they should indicate how many generations apart the lines are and include some data from littermates to increase confidence that the WT values are indeed the appropriate control comparison.

For each experiment, we only used age and gender-matched controls from the same cohort. All knockout animals were obtained from Taconic and maintained on a C57BL/6NTac background from Taconic. Neurogenesis is very sensitive to the age of the mice. Thus, we are unable to not compare animals of different ages or combine control animals from different cohorts or sources. The two experiments highlighted by the reviewer are one such example, where these differences in cell counts represent animals from two separate cohorts each with their own appropriate control group. The slight differences between the two experiments are a reflection of experimental variations due to difference in timing, age, experimenter and source. In particular, we would like to note that the K-/- and D-/- mice and their age-matched controls were 3 months of age and the

KD-/- mice and their age-matched controls were 2 months of age. Age of all genotypes is now clearly stated in the corresponding figure legends.

(5) More details about the samples used for the experiments should be included. When sample size is presented as n = 4, for example, the authors should explain whether there are 4 sections or 4 mice used, and whether the mice were from separate litters.

Within each mouse cohort, we used age and gender-matched littermate controls. To get enough statistical power, several breeding pairs are set up in parallel. For histological analysis, when a sample size is presented as n=4, this indicates that the analysis was performed on 3-5 hippocampal sections per mouse for a total of 4 mice per group. Each data point on the graph represents the average cell count from the 3-5 sections for that particular mouse. We updated the method section and figure legends to add clarification.

(6) On page 5, the authors state that "Together, these data suggest the influence of H2-Kb molecules in the adult hippocampal neurogenic niche is exerted during later stages of neurogenesis rather than the stem cell pool. " But, doesn't the increase in Edu+ cells in panel F, Figure 1, as well as other data in the paper indicate that H2-Kb regulates early stages of proliferation?

We agree with the reviewer that the use of the term “later” is lacking accuracy when describing the neurogenic process. The statement has been removed in the revised manuscript.

(7) Figure S4 is interpreted as demonstrating that there is no change in the % of neurons or astros in the cultures generated from WT, K-KO or D-KO mice. Yet, it looks like there might be a decrease in the # of cells from the D-KO, which would be important if true since a main conclusion from this paper is that H2-D does not play a role in adult neurogenesis. Including the actual data plotted (average value +/- sem, and the p-value) and increasing sample size for at least the controls in this particular experiment would determine if this trend is real. While it could be argued that increasing sample size may not be warranted due to power analysis, the actual variability in the experiment indicates that additional samples should be included to make the conclusion that H2-D does not affect proliferation.

The revised manuscript now includes bar graphs with individual data points to reflect the mean, SEM and variability more easily. Additionally, we repeated the spontaneous differentiation experiment in adult NSPCs infected with lentivirus encoding shRNA targeting either H2-K1 or H2-D1 and did not detect significant differences compared to control conditions despite an increased sample size.

(8) The approach used to determine whether cells express H2-K, or not, in Figure 2 seems potentially arbitrary. The authors should include a justification for selecting the threshold of 5 CPM as positive or negative for expression.

The reviewer raises an important point. In order to test the impact of different thresholds for H2-K1 expression on the observation that *H2-K1* expression and cell cycle (based on cell-cycle score) tend to be two exclusive processes in the single-cell dataset, we calculated the odds ratio for different thresholds of *H2-K1* (Reviewer figure 1 below, odds ratio <1 suggests trend towards exclusivity). The odds ratio in this case is defined as ratio of cycling versus non-cycling K1-positive cells divided by cycling versus non-cycling K1-negative cells, i.e. (K1+Cycling / K1+ Non-Cycling) / (K1-Cycling / K1- Non-Cycling). A ratio of less than 1 indicates that K1-positive cells are less likely to be cycling, and this hypothesis is supported across the range of CPM thresholds we

tested (from 0 to 20). Due to the potentially arbitrary choice of the threshold, we have adjusted our language of the interpretation of the findings in the results section of the manuscript.

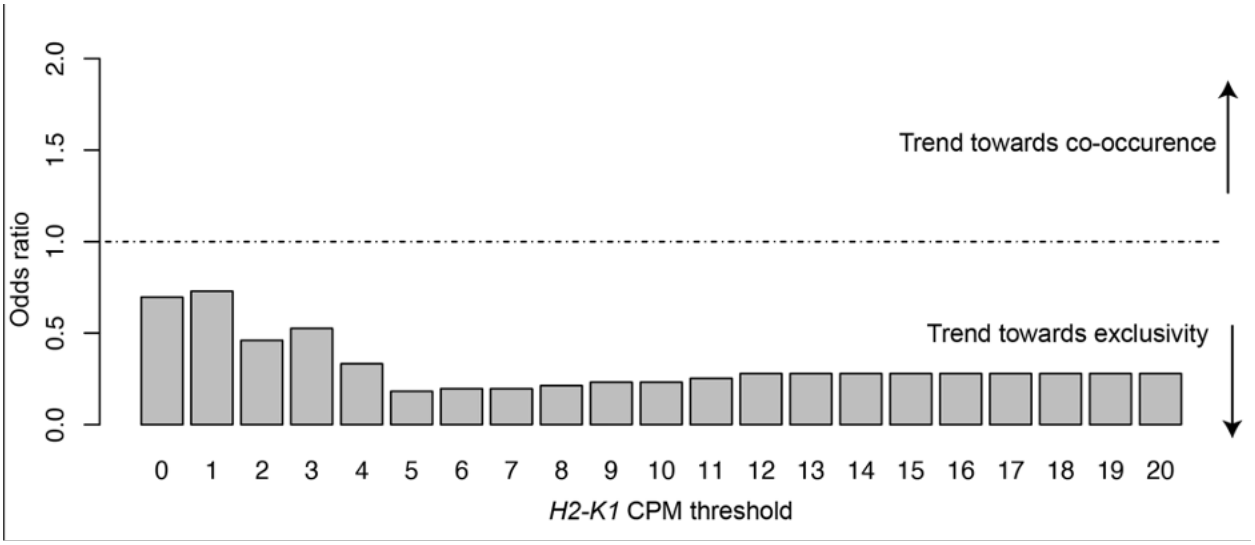

Reviewer figure 1. Odds ratio (*y-axis*, K1+Cycling / K1+ Non-Cycling) / (K1-Cycling / K1- Non-Cycling) for different thresholds of *H2-K1* expression (*x-axis*). Values below 1 indicate exclusivity, above 1 co-occurrence.

(9) On page 6, the authors state that "These bioinformatics data provide evidence that H2-Kb regulates NSPC proliferation in a cell-intrinsic manner, potentially by impacting the cell cycle." However, it is unclear why they conclude that this is cell-intrinsic.

We agree with the reviewer that the question whether this is cell intrinsic or not cannot be derived from the bio-informatic single cells analysis of NPCs and have adapted the statement accordingly in the revised manuscript.

(10) Figure 3, panel B. The graph appears to show a significant decrease in Fgfr1 expression in H2-D KO cells that is not indicated by an asterisk. This decrease would be important to note, if true, given the clear conclusion by the authors that H2-D does not regulate proliferation.

The Fgfr1 expression in wildtype, K and D knockout NSPCs in the previous version of the Manuscript was compared using a One-Way ANOVA with a Dunnett's multiple comparisons test where both K-/- and D-/- were compared to the WT control group. This analysis was not significant for the WT and D-/- comparison. Additionally, we have now measured Fgfr1 expression in WT adult NSPCs following viral-mediated abrogation and overexpression of H2-K1 or H2-D1, and observe that H2-K1, but not H2-D1, bi-directionally alters the expression levels of Fgfr1 (Figure 3E). We conclude that in these experimental settings, H2-D1 does not appear to regulate Fgfr1 expression in NSPCs.

(11) Figure 3, panels D and E. Although the FGFR inhibitor largely rescues the sh-K-induced increase in proliferation in adult neurons, it is less effective in postnatal neurons. The authors should discuss potential reasons why this is the case.

This is an interesting observation. It is likely that the postnatal versus adult NSPCs display distinct sensitivity to growth factors due to differences in their respective developmental stages. However, we would like to highlight that the overall directionality of the changes in proliferation induced by decreased H2-K1 expression remain the same between postnatal and adult-derived NSPCs.

(12) The authors interpret the result in Figure 3 as showing that "H2-Kb molecules negatively regulate NSPC proliferation by inhibiting FGFR-mediated signaling." Yet, the authors do not show this directly. To do so, they would need to increase H2Kb levels, show a decrease in FGFR signaling and in proliferation, and then rescue both by normalizing FGFR signaling.

To complement the pharmacological approach used in the previous version of the manuscript, we performed necessity and sufficiency experiments in adult NSPCs using viral-mediated genetic manipulations of Fgfr1. We generated novel lentiviral constructs encoding shRNA targeting Fgfr1 or overexpressing Fgfr1 under the control of the mouse Nestin promoter. We now demonstrate that reducing the increase in Fgfr1 expression observed in NSPCs after H2-K1 abrogation mitigates the effect on cell proliferation (Figure 3G). Conversely, mimicking the increase in Fgfr1 expression observed in NSPCs following H2-K^b abrogation by viral-mediated Fgfr1 overexpression increased cell proliferation (Figure 3H). We believe these new data provide additional evidence for a link between H2-K1 and Fgfr1 in regulating NSPC proliferation.

Minor:
(1) Fig1. Legend- refers to S2, should be reference to S1.

We corrected the label of the corresponding figure legend.

(2) Fig2. Panel F X-axis should be labeled. J) Provide the number of cells in each group somewhere (H2-K1 positive, H2-K1 negative).

The label now states "*Log2 mean expression WT*" in Fig S7B and the cell counts were added into the corresponding figure legend.

(3) Fig3. B) make clear in axis label that this is RNA.

The gene names are now italicized throughout all figures when referring to RNA.

(4) Typo "ribosomal Proteign" in figure legend.

The typo was corrected.

Methods
(5) AG 99 is referenced in inhibitor experiments but it is unclear in what experiments it is used.

The reference to AG 99 was removed from the revised manuscript.

(6) Methods include references to western blot quantification, but none is presented in the paper

We now include quantifications of the updated Western blot.

(7) Details are missing about how long after lentiviral injection for knockdown experiments BRDu was injected and animals perfused.

Experimental details have now been included in the revised method section.

(8) C57 Bl6N(Tac) and Bl6J are both listed in methods. Which strain was a control for which experiments?

All knockout animals were obtained from Taconic and maintained on a C57BL/6NTac background from Taconic. Since the H2-K1 and H2-D1 genes are located in relatively close-proximity on mouse chromosome 17, the KD-/- double-knock out mutations are co-inherited. Thus, the KD double knock-out line is maintained separately from the single knock out mouse lines. For each experiment, we only used age and gender-matched controls from the same cohort. All stereotaxic experiments using the lentiviral-mediated shRNA and overexpression constructs were performed in C57Bl6J mice from JAX. On each surgery day, equal number of animals were injected with the different viruses.
