# Supplementary material for: MHC class I H2-Kb negatively regulates neural progenitor cell proliferation by inhibiting FGFR signaling
Source: PLoS Biol. 2021 Jun 28;19(6):e3001311. doi: 10.1371/journal.pbio.3001311 (PMC8270425; doi:10.1371/journal.pbio.3001311)
Supplement: Attachment — Submitted filename: Lin_PLOSBIO_Response-to-Reviewers_060221_SV.pdf [file pbio.3001311.s015.pdf]

Response to reviewer comments of manuscript “MHC Class I H2-K^b Negatively Regulates Neural Progenitor Cell Proliferation by Inhibiting FGFR Signaling” (PBIOLOGY-D-20-02289R2)

We would like to thank the reviewers for their helpful and constructive comments throughout the peer review process. Their input has helped us to significantly strengthen the functional link between MHC class I molecules, FGFR signaling and neural progenitor cell proliferation.

Please find below a point-by-point discussion to the reviewers' comments. In our reply, the questions/comments are in black and our replies are in blue font.

Reviewer #1: The authors followed my recommendations and provided convincing gain-of-function studies, that nicely complement the loss-of-function approach. Also, the strengthened the link between H2-K1 and Fgfr1 expression with new and convincing data. I have no further critique.

Reviewer #2: The authors have addressed all my concerns.

Reviewer #3: In their resubmission of PBIOLOGY-D-20-02289R1, the authors present a thorough response to all of the critiques and the manuscript is much improved. The only experiment they did not perform was to determine if FGFR overexpression rescues the decrease in proliferation caused by H2Kb overexpression. They have the constructs to perform this experiment, so it's unclear why they couldn't complete it. That being said, they do show strong evidence that H2-K1 and Fgfr1 signaling is linked and regulates NSPC proliferation. That is a novel and important finding and I am convinced that the manuscript is now strong enough for publication.

We thank all reviewers for their positive responses and are pleased to see that their comments were satisfactorily adressed. While we did not overexpress Fgfr1 in the context of H2Kb overexpression, as noted by the reviewers, the new data provided establishes a strong functional link between H2-Kb, Fgfr signaling, and NSPC proliferation. We are enthusiastic about the reviewer's support of our manuscript for publication in *PLOS Biology*.
